# MicroRNA-19a Targets Fibroblast Growth Factor-Inducible Molecule 14 and Prevents Tubular Damage in Septic AKI

**DOI:** 10.1155/2020/2894650

**Published:** 2020-07-01

**Authors:** Jun Hong, Bang-Chuan Hu, Liang Xu, Yang Zheng, Zi-Qiang Shao, Run Zhang, Xiang-Hong Yang, Ren-Hua Sun, Shi-Jing Mo

**Affiliations:** Department of Intensive Care Unit, Zhejiang Provincial People's Hospital, People's Hospital of Hangzhou Medical College, Hangzhou, 310014 Zhejiang, China

## Abstract

Fibroblast growth factor-inducible molecule 14 (Fn14) plays a principal role in triggering tubular damage during septic acute kidney injury (AKI). Here, we explore the mechanism underlying Fn14 deregulation in septic AKI. We identify Fn14 as a bona fide target of miR-19a, which directly binds to 3′ UTR of Fn14 for repression independent of cylindromatosis (CYLD), the deubiquitinase (DUB) downstream of miR-19a, and thereby antagonizes the LPS-induced tubular cell apoptosis. Genetic ablation of Fn14, but not of CYLD, abolishes the ability of miR-19a to antagonize the tubular apoptosis by lipopolysaccharide (LPS). In mice, systemic delivery of miR-19a confers protection against septic AKI. Our findings implicate that miR-19a may serve as a promising therapeutic candidate in the prevention of septic AKI.

## 1. Introduction

Septic acute kidney injury (AKI) is the most thorny organ failure (OF) and common cause of mortality in the intensive care unit (ICU), with the simultaneous presence of both sepsis-3.0 and Kidney Disease: Improving Global Outcomes (KDIGO) criteria as the clinical diagnosis [1, 2]. The main pathological features of septic AKI include deleterious endotoxemia resulting from systemic inflammatory response syndrome (SIRS), abnormal serum creatinine (Scr), and blood urea nitrogen (BUN) levels due to tubular damage. Although implementation of antibiotics, fluid bolus (FB), and renal replacement (RR) therapies has shown benefit for preventing septic AKI, there is no satisfactory therapy in accelerating recovery from septic AKI to date. Accordingly, development of an innovative therapeutic strategy to improve the prognosis of patients with septic AKI might be urgent.

Fibroblast growth factor-inducible molecule 14 (Fn14, encoded by the TNFRSF12a gene), a type I transmembrane protein and member of the fibroblast growth factor-inducible gene family, functions as the unique receptor of tumor necrosis factor-like weak inducer of apoptosis (TWEAK) [3]. Fn14 is ubiquitously expressed in various tissues and has been reported to participate in multiple physiological and pathological processes including inflammation, ischemia-reperfusion (IR) injury, cell proliferation, differentiation, and apoptosis [4]. Fn14 expression is relatively low in normal healthy tissues, while it could be rapidly activated in response to stress stimuli, which represents one of the main regulatory mechanisms for evoking inflammation or apoptosis. Activation of Fn14 by recombinant TWEAK (rTWEAK) mediates tubular damage and nephrotoxicity upon cyclosporin A (CsA) exposure [5]. Fn14 deficiency attenuates kidney IgG deposition, decreases inflammatory cell infiltration, and protects mice from developing lupus nephritis [6]. Our previous study demonstrated that lipopolysaccharide (LPS) triggers tubular cell apoptosis as a result of Fn14 stabilization, and blockade of Fn14 is sufficient to improve kidney function and prevent septic AKI in mice [7]. However, the mechanism underlying Fn14 deregulation in tubular damage during septic AKI remains largely unknown.

Being able to degrade and translationally inhibit the target gene through binding to the complementary sequences in the 3′ UTR of messenger RNAs (mRNAs), microRNA (miRNA) clusters have been implicated in the development and progression of lethal sepsis or AKI [8–10]. Given the critical role of Fn14 in septic AKI according to our previous findings, it is of particular interest to identify miRNA that interferes with Fn14 signaling, particularly those engaged in tubular damage. In this study, we provide evidence to identify Fn14 as a direct downstream target of miR-19a, which antagonizes tubular apoptosis following LPS stimuli in a CYLD-independent fashion. Systemic delivery of miR-19a made mice resistant to septic AKI, supporting the therapeutic value of restoring miR-19a expression in the management of clinical patients with septic AKI.

## 2. Materials and Methods

### 2.1. Cell Culture

Murine proximal tubular epithelial MCT and macrophage-like RAW 264.7 cells were maintained in RPMI 1640 medium (Gibco, Carlsbad, USA) or Dulbecco's Modified Eagle's Medium (DMEM) supplemented with 10% fetal bovine serum, 100 U/mL penicillin, and 100 *μ*g/mL streptomycin at 37°C under 95% humidity with 5% CO_2_ as previously described [7].

### 2.2. Transfection

miR-19a mimics and the negative controls of lin4 miRNA mimics were as previously indicated [11] and transfected at a final concentration of 100 nM in the cells using HiPerFect Transfection Reagent (QIAGEN, Hilden). Small interfering RNA (siRNA) duplex oligonucleotides targeting Fn14 and CYLD were as previously described [7, 11] and transfected into cells using Lipofectamine 2000 (Invitrogen, Carlsbad, CA, USA) according to the manufacturer's recommendations. Cells were harvested for subsequent experiments after 48 hours of transfection.

### 2.3. Cell Viability Assay

Cell viability was measured by a 3-(4,5-dimethylthiazol-2-yl)-2,5-diphenyltetrazolium bromide (MTT) reduction assay as described previously [12–14].

### 2.4. DEVDase Activity Assay

DEVDase activity assay was performed using caspase-3 activity detection kit (Bestbio, Shanghai, China) as described in previous publications [12–14].

### 2.5. TUNEL Assay

Terminal deoxynucleotidyl transferase-mediated dUTP-biotin nick end labeling (TUNEL) assay was carried out using Roche's In Situ Cell Death Detection Kit (Roche, Germany) according to the manufacturer's guidelines as reported previously [15]. The detailed information is indicated in Supplemental Materials and Methods.

### 2.6. Dual-Luciferase Reporter (DLR) Assay

Cells were seeded in triplicate in 24-well plates and allowed to settle for 24 hours. Indicated plasmids plus 1 ng pRL-TK *Renilla* plasmid were transfected into the cells using Lipofectamine 2000 Reagent (Life Technologies). Forty-eight hours after transfection, Dual-Luciferase Reporter Assay (Promega) was performed according to the manufacturer's instructions, as previously described [14, 15]. The 3′ UTR regions of wild-type and mutant Fn14 were amplified by PCR using the following primers: Fn14-WT: 5′-CGAGCTCGAAGCCTCAATCTGGGTCACAA-3′ (forward) and 5′-CCCGGGG GCATTATAGCCCCTCCGAGT-3′ (reverse). Fn14-Mut: 5′-CGAGCTCGAACTCGGAGGGGCTATAATGC-3′ (forward) and 5′-CCCGGGGGGAGATGGTTGTTTCCGTGT-3′ (reverse).

### 2.7. Real-Time Quantitative PCR (RT-qPCR)

Detection of mRNA was performed as described previously [7, 14]. The detailed information is indicated in Supplemental Materials and Methods.

### 2.8. Cellular Ubiquitination Assay

Cellular ubiquitination assay was performed according to a standard method, as described previously [7, 14, 15]. The detailed information is indicated in Supplemental Materials and Methods.

### 2.9. Western Blotting

Western blotting analyses were performed as described previously with some modifications [7, 13, 15]. The detailed information is indicated in Supplemental Materials and Methods.

### 2.10. Cecal Ligation and Puncture (CLP) and Endotoxemia Experiments

All animal studies were conducted with the approval of the Hangzhou Medical College Institutional Animal Care and Use Committee and were performed in accordance with established guidelines. The detailed procedures for cecal ligation and puncture (CLP) experiments were described previously [7]. For delivery in vivo, the miR-19a mimics were incubated with Invivofectamine (IVF3005, Thermo Fisher Scientific, Inc., Waltham, MA, USA) and then injected into the CLP mice through the tail veins at the indicated times. To generate the endotoxemia model, mice received an intraperitoneal infusion of LPS (*E. coli* 0111: B4, 10 mg/kg, Sigma) and then delivered with miR-19a mimics or negative control (NC). Serum samples were collected and stored at -20°C before analysis.

### 2.11. Immunofluorescence for Cleaved Caspase-3 Detection

After being fixed with 4% paraformaldehyde in PBS at room temperature, permeabilized in 0.5% Triton X-100, and washed twice in PBS, cells plated in six-well plates were incubated with 5% bovine serum albumin to block nonspecific binding and then incubated with anticleaved caspase-3 antibody (1 : 500, Cell Signaling Technology) overnight at 4°C, followed by incubation with Alexa Fluor 594 goat anti-rabbit IgG (1 : 500) for 1 h at 37°C. The nuclei were stained with 5 *μ*g/mL DAPI for 15 min and viewed with an IX71 fluorescence microscope.

### 2.12. Immunohistochemical Staining

Immunohistochemical staining was carried out according to the previous publication [7]. The detailed information is indicated in Supplemental Materials and Methods.

### 2.13. Statistical Analysis

Comparison between two groups was determined by unpaired two-tailed Student's *t*-tests, and *P* values less than 0.05 were considered statistically significant. Statistical comparisons in multigroup analysis were assessed by one-way ANOVA. All data are shown as means ± standard deviation for at least three independent experiments unless otherwise indicated.

## 3. Results

### 3.1. Fn14 Is a Bona Fide Target of miR-19a

**Figure 1:**
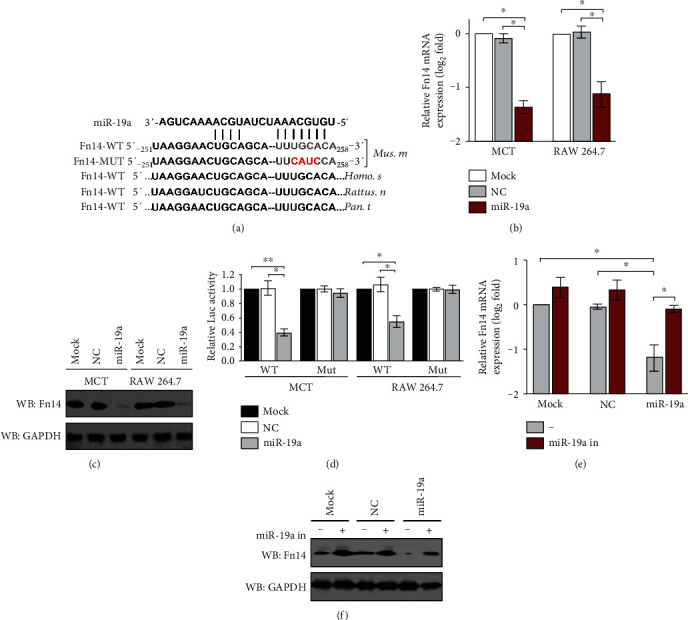
Fn14 is a bona fide target of miR-19a. (a) Predicted miR-19a target sequence in wild-type Fn14-3′ UTR (Fn14-WT) among mammalian orthologs and mutant Fn14-3′ UTR containing mutated nucleotides in the miR-19a seed-pairing region (Fn14-Mut). (b) RT-qPCR evaluating levels of Fn14 mRNA expression in murine MCT and RAW 264.7 cells transfected with mock (Mock), negative control (NC), and miR-19a mimics (miR-19a) for 48 h, respectively. ^∗^*P* < 0.05, one-way ANOVA, post hoc comparisons, and Tukey's test. (c) Western blotting measuring abundance of Fn14 protein in murine MCT and RAW 264.7 cells transfected with mock (Mock), negative control (NC), and miR-19a mimics (miR-19a) for 48 h, respectively. GAPDH was used as the internal control of cytoplasmic extractions. (d) Luciferase assays of Fn14-3′ UTR activity in murine MCT and RAW 264.7 cells cotransfected with WT or Mut Fn14-3′ UTR reporter constructs and mock (Mock) and negative control (NC) as well as miR-19a mimics (miR-19a) for 48 h, respectively. ^∗^*P* < 0.05 and ^∗∗^*P* < 0.01, one-way ANOVA, post hoc comparisons, and Tukey's test. (e) RT-qPCR assessing abundance of Fn14 mRNA expression in murine MCT cells with mock (Mock) and negative control (NC) as well as miR-19a mimic (miR-19a) transfection in the presence or absence of miR-19a inhibitor (miR-19a in) cotransfection, respectively. ^∗^*P* < 0.05, one-way ANOVA, post hoc comparisons, and Tukey's test. (f) Western blotting analyses comparing levels of Fn14 protein expression in murine MCT cells with mock (Mock) and negative control (NC) as well as miR-19a mimic (miR-19a) transfection in the presence or absence of miR-19a inhibitor (miR-19a in) cotransfection, respectively.

Analysis using publicly available algorithms (TargetScan and miRanda) predicted Fn14 as a direct downstream target of miR-19a, which contains a conserved binding site of 3′ UTR in Fn14 mRNA among mammalian orthologs (Figure 1(a)). In an attempt to dissect whether miR-19a influences Fn14 expression, we transfected murine proximal tubular epithelial MCT cells with miR-19a mimics and examined the levels of Fn14 mRNA and protein expression using real-time quantitative reverse transcriptase-polymerase chain reaction (RT-qPCR) and western blotting analyses, respectively. Data from Figure 1(b) showed that miR-19a downregulated the mRNA expression of Fn14 (*P* < 0.05), which kinetically correlated with a lower abundance of Fn14 protein during the course of the gain-of-function experiments (Figure 1(c)). Similar results in both cases were recapitulated in murine macrophage-like RAW 264.7 cells (*P* < 0.05, Figures 1(b) and 1(c)), indicating that the repressive role of miR-19a in Fn14 is a common feature not restricted to cell lineage. These findings are in line with a luciferase reporter assay where a luciferase reporter containing the Fn14-3′ UTR with wild-type or mutant miR-19a seed-pairing region was transfected into the mock-, negative control- (NC-), or miR-19a-expressed cells, showing that miR-19a, instead of mock or NC, remarkably reduced the activities of the luciferase reporter fused to the wild-type, but not the mutant, Fn14-3′ UTR in all cells tested (*P* < 0.05 and *P* < 0.01, Figure 1(d)). These results strongly suggest that Fn14 is a bona fide target of miR-19a.

We next determined the possibility of off-target impact of miR-19a on Fn14. To test this, we introduced the miR-19a inhibitor (miR-19a in) into the miR-19a mimic-transfected MCT cells and then examined the resultant effects. As shown in Figures 1(e) and 1(f), MCT cells with miR-19a mimic transfection had decreased Fn14 mRNA and protein levels which could be rescued by concurrent expression of the miR-19a inhibitor (*P* < 0.05), which by itself modestly upregulated Fn14 mRNA and protein expression, suggesting that the miR-19a-mediated Fn14 repression was abrogated by inhibition of miR-19a.

Intriguingly, LPS stimuli downregulated levels of miR-19a mRNA expression in both MCT and RAW 264.7 cells, the effects of which could be abolished by a pan-HDAC inhibitor trichostatin A (TSA) or a selective HDAC3 inhibitor RGFP966 (RGFP), indicating that histone deacetylase 3 (HDAC3) might, at least in part, engage in the LPS-inducible downregulation of miR-19a (*P* < 0.001 and *P* < 0.01, Figure S1).

### 3.2. Repression of Fn14 and CYLD by miR-19a Is Independent of Each Other

Our previous study demonstrated that miR-19a represses CYLD expression [11]. We thus surmised whether CYLD is involved in the miR-19a-mediated Fn14 repression. Unexpectedly, ectopic expression of CYLD in MCT cells had negligible effects on the mRNA levels of Fn14 but slightly enhanced its protein expression (Figure S2(a) and S2(b)), presumably due to the dismantled K48-linked Fn14 polyubiquitination (Figure S2(c)). However, miR-19a still downregulated the mRNA and protein expression of Fn14 in the CYLD-deficient MCT cells (*P* < 0.05, Figure S2(d-f)). Vice versa, CYLD could also be downregulated by miR-19a in the Fn14-deficient cells as in the control cells (data not shown), implying that miR-19a represses Fn14 and CYLD independent of each other.

### 3.3. miR-19a Inhibits the LPS-Induced Tubular Damage

**Figure 2:**
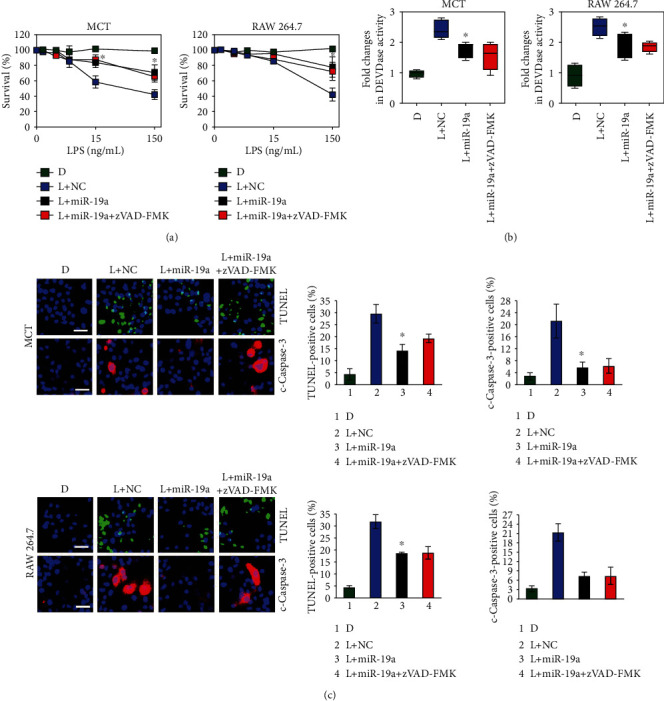
miR-19a inhibits the LPS-induced tubular apoptosis. (a) Murine MCT and RAW 264.7 cells with negative control (NC) transfection, miR-19a mimic (miR-19a) transfection, or miR-19a mimic (miR-19a) transfection plus zVAD-FMK treatment were separately stimulated with the indicated concentrations of LPS (L) for 72 h, and the cell viabilities were measured by MTT assay. ^∗^*P* < 0.05 versus L+NC, one-way ANOVA, post hoc comparisons, and Tukey's test. D: DMSO. (b) DEVDase activity of murine MCT and RAW 264.7 cells with negative control (NC) transfection, miR-19a mimic (miR-19a) transfection, or miR-19a mimic (miR-19a) transfection plus zVAD-FMK treatment in the presence or absence of 150 ng/mL LPS (L) stimuli for 72 h. ^∗^*P* < 0.05 versus L+NC, one-way ANOVA, post hoc comparisons, and Tukey's test. (c) Representative pictures and quantification from TUNEL- and cleaved caspase-3- (c-caspase-3) staining assay of murine MCT and RAW 264.7 cells with negative control (NC) transfection, miR-19a mimic (miR-19a) transfection, or miR-19a mimic (miR-19a) transfection plus zVAD-FMK treatment in the presence or absence of 150 ng/mL LPS (L) stimuli for 72 h. ^∗^*P* < 0.05 versus L+NC, one-way ANOVA, post hoc comparisons, and Tukey's test.

Given the exclusive inhibition of Fn14 by miR-19a and our previous study showing that Fn14 contributes to the LPS-induced tubular apoptosis [7], we sought to investigate whether the apoptosis-inducible role of LPS could be impacted by miR-19a and, if so, whether this is dependent on Fn14. To this end, we stimulated the miR-19a-expressed MCT and RAW 264.7 cells with different concentrations (from 0 to 150 ng/mL) of LPS for 72 h and then performed the MTT assay to measure cell viability (Figure S3(a) and S3(b)).  Figure 2(a) showed that LPS dose-dependently decreased the survival of both cells, which could be successfully rescued by miR-19a (*P* < 0.05). In accordance with these observations, LPS stimuli elevated the magnitude of DEVDase activities and the percentage of TUNEL- as well as cleaved caspase-3-positive apoptosis cells in the caspase-3 activity assay, TUNEL, and cleaved caspase-3 staining analyses, respectively, yet forced expression of miR-19a abrogated the LPS-induced apoptosis as reflected by the data that the cells expressing miR-19a had diminished magnitude of DEVDase activities (*P* < 0.05) and percentage of TUNEL- as well as cleaved caspase-3-positive staining (*P* < 0.05) in comparison to the cells expressing NC upon LPS stimuli (Figures 2(b) and 2(c)). Nevertheless, treatment with benzyloxycarbonyl-Val-Ala-Asp-fluoromethylketone (zVAD-FMK), a pancaspase inhibitor, did not further potentiate the capacity of miR-19a to enhance survival in the LPS-stimulated cells (Figures 2(a)–2(c)), indicating that miR-19a and caspase inhibition might act in the same way to block tubular damage during LPS stimuli.

### 3.4. Fn14 Contributes to the LPS-Induced Tubular Damage in a miR-19a-Dependent Manner

**Figure 3:**
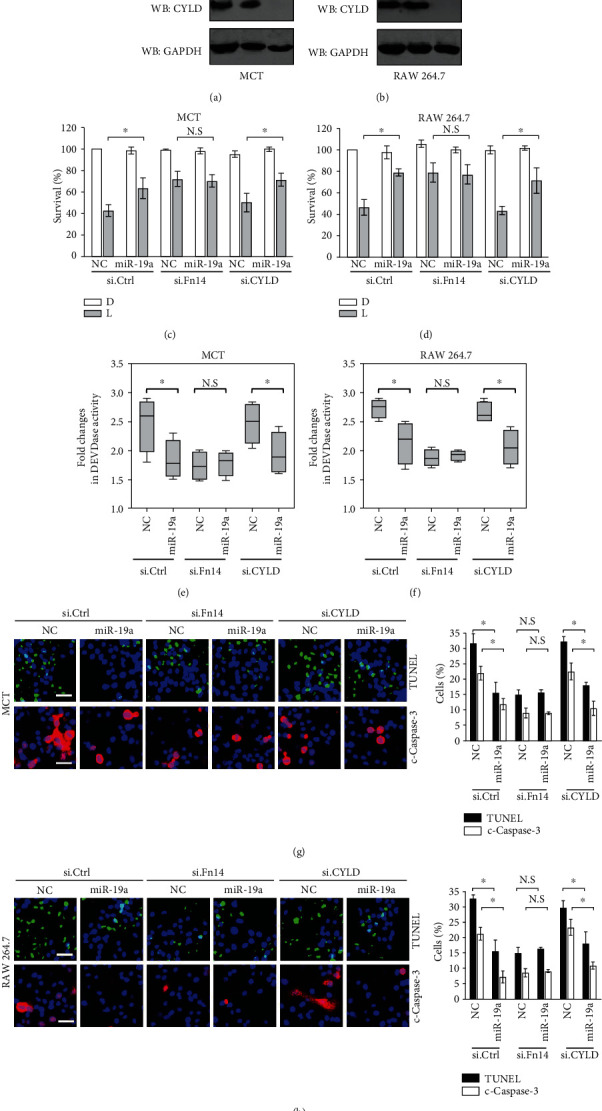
Fn14 contributes to the LPS-induced tubular apoptosis in a miR-19a-dependent manner. (a, b) Western blotting analyses detecting the abundance of Fn14 and CYLD protein in MCT (a) and RAW 264.7 (b) cells with control (Ctrl) siRNA, Fn14 siRNA (si.Fn14), or CYLD siRNA (si.CYLD) transfection, respectively. (c, d) MTT assay measuring cell viabilities of negative control- (NC-) or miR-19a mimic- (miR-19a-) transfected MCT (c) and RAW 264.7 (d) cells exposed to 150 ng/mL LPS (L) for 72 h with control (Ctrl) siRNA, Fn14 siRNA (si.Fn14), or CYLD siRNA (si.CYLD) cotransfection, respectively. ^∗^*P* < 0.05, one-way ANOVA, post hoc comparisons, and Tukey's test. D: DMSO. N.S: not significant. (e, f) DEVDase activity of negative control- (NC-) or miR-19a mimic- (miR-19a-) transfected MCT (e) and RAW 264.7 (f) cells exposed to 150 ng/mL LPS (L) for 72 h with control (Ctrl) siRNA, Fn14 siRNA (si.Fn14), or CYLD siRNA (si.CYLD) cotransfection, respectively. ^∗^*P* < 0.05, one-way ANOVA, post hoc comparisons, and Tukey's test. (g, h) Representative pictures and quantification from TUNEL- and cleaved caspase-3- (c-caspase-3-) staining assay of negative control- (NC-) or miR-19a mimic- (miR-19a-) transfected MCT (g) and RAW 264.7 (h) cells exposed to 150 ng/mL LPS (L) for 72 h with control (Ctrl) siRNA, Fn14 siRNA (si.Fn14), or CYLD siRNA (si.CYLD) cotransfection, respectively. ^∗^*P* < 0.05, one-way ANOVA, post hoc comparisons, and Tukey's test.

We next wondered whether Fn14, which ultimately leads to tubular damage, could abrogate the apoptosis-resistant phenotypes of miR-19a during LPS stimuli. To address this issue, we transfected the miR-19a-expressed MCT and RAW 264.7 cells with small interfering RNA (siRNA) targeting Fn14 (si.Fn14) (Figure S3(c), S3(d) and Figures 3(a) and 3(b)) and then stimulated them with LPS. As shown in Figures 3(c) and 3(d), miR-19a markedly increased the proportion of surviving cells upon LPS stimuli but failed to do so when concomitantly silencing Fn14 under the same conditions (*P* < 0.05). In agreement with these observations, the miR-19a-expressed cells with LPS stimulation had statistically significant lower magnitude of DEVDase activities (*P* < 0.05) and percentage of TUNEL- as well as cleaved caspase-3-positive staining (*P* < 0.05) than the NC-expressed cells that were stimulated with LPS in the presence of si.Ctrl transfection, while this did not occur in the presence of si.Fn14 transfection (Figures 3(e)–3(h)). Identical silencing of CYLD had minimal effects on the apoptosis-resistant ability of miR-19a in the LPS-stimulated cells (Figures 3(a)–3(h)). These results together suggest that Fn14 deficiency is specific and instrumental for the miR-19a-dependent tubular apoptosis resistance against LPS.

### 3.5. miR-19a Protects Mice from Septic AKI

**Figure 4:**
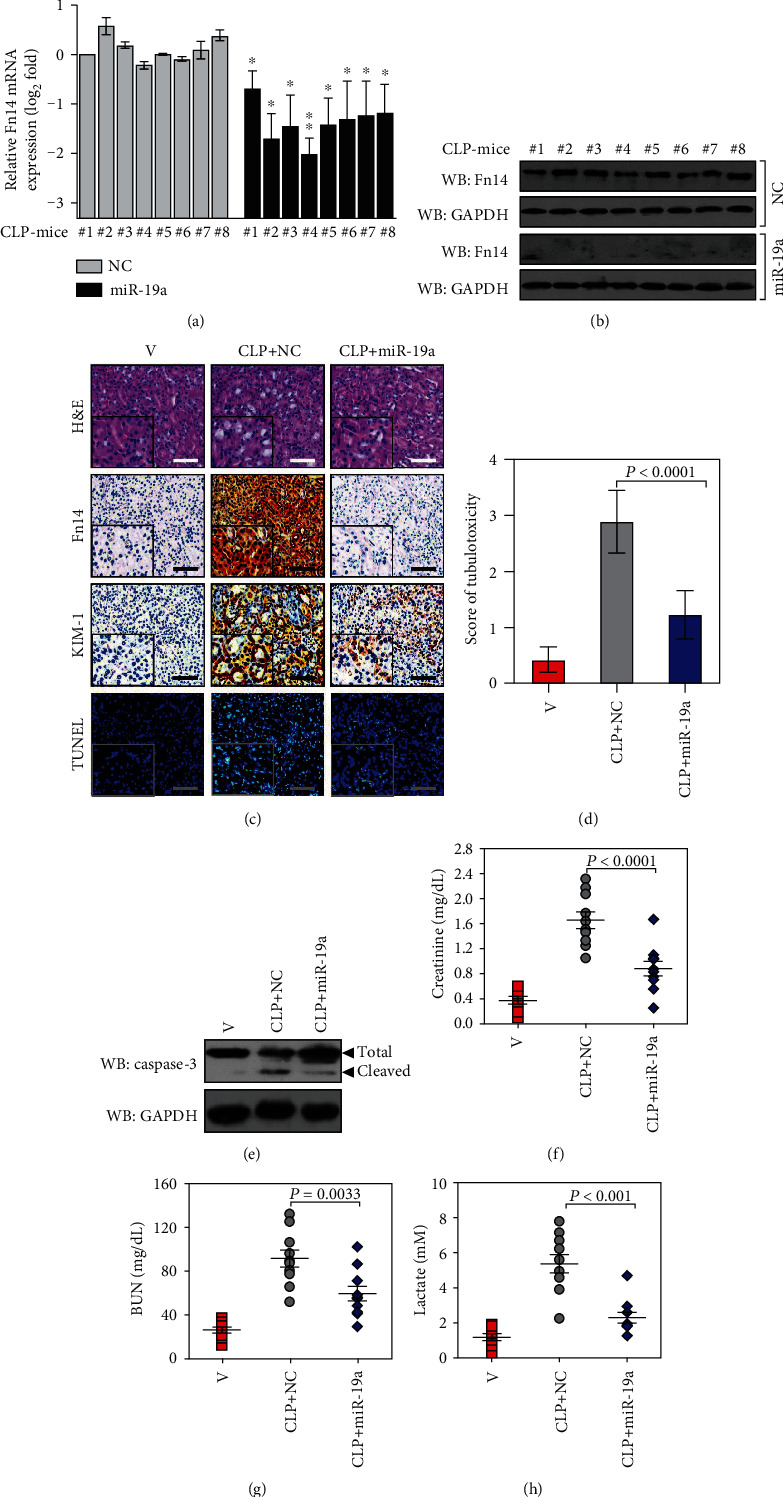
miR-19a protects mice from septic AKI. (a) RT-qPCR analysis examining Fn14 mRNA expression in kidney tissues from CLP-treated mice with negative control (NC) or miR-19a mimic (miR-19a) delivery (*n* = 8). ^∗^*P* < 0.05 and ^∗∗^*P* < 0.01 versus NC, one-way ANOVA, post hoc comparisons, and Tukey's test. (b) Western blotting analyses comparing the levels of Fn14 protein in kidney tissues from CLP-treated mice with negative control (NC) or miR-19a mimic (miR-19a) delivery (*n* = 8). (c) Representative H&E and IHC as well as TUNEL staining images for Fn14, KIM-1, and TUNEL in kidney tissues from CLP-treated mice with negative control (NC) or miR-19a mimic (miR-19a) delivery. (d) Tubular damage score of kidney tissues from CLP-treated mice with negative control (NC) or miR-19a mimic (miR-19a) delivery (*n* = 8). One-way ANOVA, post hoc comparisons, and Tukey's test. V: vehicle. (e) Western blotting analyses detecting the cleavage of caspase-3 in kidney tissues from CLP-treated mice with negative control (NC) or miR-19a mimic (miR-19a) delivery. (f–h) Serum creatinine (f), blood urea nitrogen (BUN) (g), and lactate (h) levels in kidney tissues from CLP-treated mice with negative control (NC) or miR-19a mimic (miR-19a) delivery (*n* = 8). One-way ANOVA, post hoc comparisons, and Tukey's test. V: vehicle.

The resistant phenotypes of miR-19a in LPS-induced tubular apoptosis mediated by Fn14 repression raise the possibility that miR-19a is likely to protect mice from septic AKI. To approach this, we utilized cecal ligation and puncture (CLP) to generate lethal sepsis in C57BL/6 mice and delivered them with miR-19a through the tail veins at 24+ hours following the onset of lethal sepsis for a further three days. In vivo delivery of miR-19a, but not NC, significantly downregulated the levels of Fn14 mRNA and protein expression in kidney tissues from the CLP-treated mice, which coincide with our in vitro data concluding that Fn14 is a bona fide target of miR-19a (*P* < 0.05, Figures 4(a) and 4(b)). As determined by H&E staining, the CLP-treated mice developed kidney disease with characteristic features of AKI, including loss of epithelial brush border, tubular epithelial vacuolization, and epithelial desquamation, the phenotypes that did not occur after miR-19a delivery (*P* < 0.0001, Figures 4(c) and 4(d)). The percentage of positive staining in kidney injury molecule-1 (KIM-1), an indicative of renal injury, and TUNEL, an indicative of apoptosis, was also robustly reduced in kidney tissues from CLP-treated mice with miR-19a delivery in contrast to those in kidney tissues from CLP-treated mice without (Figure 4(c)). The inhibitory role of miR-19a in septic AKI was further evidenced by the biochemical detection showing that CLP-treated mice displayed increased levels of cleaved caspase-3 (Figure 4(e)), serum creatinine (Scr) (*P* < 0.0001, Figure 4(f)), blood urea nitrogen (BUN) (*P* = 0.0033, Figure 4(g)), and lactate (*P* < 0.001, Figure 4(h)) in contrast to their littermates, the phenotypes that could be counteracted by miR-19a delivery. Similar results were obtained from AKI elicited by the LPS-inducible endotoxemia (Figure S4). These findings support the concept that miR-19a serves as a promising therapeutic target to protect mice from septic AKI.

## 4. Discussion

Albeit considerable progress in clinical management in the past decade, septic AKI remains a life-threatening OF derived from severe infections, which is often accompanied by SIRS, hypertension, anemia, shock, and poor prognosis [16]. Gram-negative bacteria such as *Acinetobacter baumannii* are the most frequently isolated pathogens in septic AKI, with LPS being the major outer membrane component and the highly conserved set of molecular structures so-called pathogen-associated molecular patterns (PAMPs) that could be recognized by PAMP receptors (e.g., NOD-like receptors, RIG-I-like receptors, and Toll-like receptors) within the innate immune system [17, 18]. Despite the intrinsic nature of Fn14 with respect to tubular damage during septic AKI having been documented in our previous study [7], the mechanism underlying Fn14 deregulation in this pathogenesis is still undefined. We hypothesized that certain miRs might function as an “inflammatory sensor” in response to LPS stimuli that negatively regulate Fn14, which would then direct tubular damage.

Our current study uncovers an unexpected role of miR-19a as an upstream inhibitor of Fn14 by targeting the 3′ UTR of Fn14 mRNA. The inhibitory roles of miR-19a in Fn14 disappeared in the presence of the miR-19a inhibitor and may not depend on the CYLD-associated Fn14 deubiquitination. We identify repression of Fn14, but not that of CYLD, as the central mechanism whereby miR-19a exerts tubular protection during septic AKI because *in vitro* cellular experiments demonstrate that miR-19a attenuates the LPS-inducible reduction in cell survival, enhancement in DEVDase activity, and elevation in TUNEL- as well as cleaved caspase-3-positive staining. The miR-19a-mediated apoptosis-resistant phenotypes can be successfully abrogated by Fn14 RNAi, indicating that miR-19a functions as a putative inhibitor of tubular apoptosis by targeting Fn14, which is necessary and sufficient to trigger tubular damage. miR-19a delivery protects mice against septic AKI and reduces their Scr, BUN, and lactate levels. Our future work will be aimed at defining the miR-19a-dependent inflammatory signal network within tubular cells, possibly offering opportunities to design and develop a small molecule activator of miR-19a for septic AKI treatment.

Accumulating evidence has suggested that miRs can play a regulatory role in sepsis and OF. For instance, let-7 belongs to an important microRNA located in a genomic locus frequently downregulated in septic acute lung injury (ALI). Overexpression of let-7 potentiates, whereas depletion of let-7 compromises, the LPS-induced inflammatory cytokine production [19]. It has been reported that miR-7-5p is required for the apoptotic inhibition of T lymphocytes and the immunosuppressive phenotypes in sepsis [20]. Low levels of serum miR-150 in sepsis patients predict unfavorable prognosis and a high ratio of organ dysfunction [21]. In addition to protein turnover, our study demonstrates that Fn14 can also be suppressed posttranscriptionally by miR-19a and that the miR-19a/Fn14 cascade determines the fate of tubular cells. Indeed, we observe higher Fn14 levels and lower abundance of miR-19a in tubular cells with LPS stimuli when compared to those in cells without LPS stimuli (data not shown). This might be a possible mechanism of how tubular cells acquire increased Fn14 levels in response to TLR4 activation.

There are, however, some limitations in our work. First, it is conceivable that other targets of miR-19a might share an equivalent role with Fn14 in accelerating septic AKI. So a better understanding of whether these targets mediate the apoptosis-refractory phenotypes of miR-19a would increase our knowledge regarding the biological characterization of septic AKI and provide new clues for developing novel therapeutic strategy. Second, based on the inhibitory role of miR-19a in Fn14 and septic AKI, the possibility that transgenic mice harboring miR-19a have alleviated tubulotoxicity during sepsis also needs to be further investigated.

In summary, we reveal an essential role of miR-19a in preventing septic AKI through repression of tubular cell apoptosis via targeting Fn14. Our findings may outline a putative mechanism of septic AKI progression and hold promise for the development of new therapeutics against septic AKI.

## Data Availability

The data used to support the findings of this study are included within the article.

## Abbreviations

Fn14: Fibroblast growth factor-inducible molecule 14
AKI: Acute kidney injury
3′ UTR: 3′ untranslated region
miRNA: MicroRNA
CYLD: Cylindromatosis
DUB: Deubiquitinase
LPS: Lipopolysaccharide
TWEAK: Tumor necrosis factor-like weak inducer of apoptosis
KDIGO: Kidney Disease: Improving Global Outcomes
SIRS: Systemic inflammatory response syndrome
DMEM: Dulbecco's Modified Eagle's Medium
CLP: Cecal ligation and puncture
KIM-1: Kidney injury molecule-1
PAMPs: Pathogen-associated molecular patterns
Scr: Serum creatinine
BUN: Blood urea nitrogen.

## Supplementary Materials

Supplementary Materials Supplemental Figure 1: histone deacetylase 3 (HDAC3) is essential for the LPS-inducible downregulation of miR-19a. (a, b) RT-qPCR detecting levels of miR-19a mRNA expression in murine MCT (a) and RAW 264.7 (b) cells stimulated with or without LPS in the presence of a pan-HDAC inhibitor trichostatin A (TSA) or a selective HDAC3 inhibitor RGFP966 (RGFP) administration. ^∗∗^*P* < 0.01 and ^∗∗∗^*P* < 0.001, one-way ANOVA, post hoc comparisons, Tukey's test. Supplemental Figure 2: repression of Fn14 and CYLD by miR-19a is independent of each other. (a) RT-qPCR determining levels of Fn14 mRNA expression in murine MCT cells transfected with empty vector (Vector) and Flag-tagged wild-type CYLD. (b) Western blotting analyses examining amount of Fn14 protein expression in murine MCT cells transfected with empty vector (Vector) and Flag-tagged wild-type CYLD. (c) Cellular ubiquitination assays detecting the levels of K48-linked polyubiquitination of Fn14 in murine MCT cells transfected with empty vector (Vector) and Flag-tagged wild-type CYLD. (d) Western blotting analyses comparing levels of CYLD protein expression in murine MCT cells with or without CYLD shRNA (sh.CYLD) transfection. (e) RT-qPCR evaluating Fn14 mRNA expression in murine MCT cells transfected with mock (Mock) and negative control (NC) as well as miR-19a mimics (miR-19a) in the presence or absence of CYLD shRNA (sh.CYLD) cotransfection. Scr: scrambled shRNA. ^∗^*P* < 0.05, one-way ANOVA, post hoc comparisons, Tukey's test. (f) Western blotting analyses assessing levels of Fn14 protein expression in murine MCT cells transfected with mock (Mock) and negative control (NC) as well as miR-19a mimics (miR-19a) in the presence or absence of CYLD shRNA (sh.CYLD) cotransfection. Supplemental Figure 3: miR-19 levels after overexpression. (a–d) RT-qPCR testing levels of miR-19a mRNA expression in murine MCT (a, c) and RAW 264.7 (b, d) cells with negative control (NC) and miR-19a mimic (miR-19a) transfection. ^∗∗∗^*P* < 0.001, two-sided Student's *t*-test. Supplemental Figure 4: miR-19a protects mice from AKI elicited by LPS-inducible endotoxemia. (a) Representative H&E and IHC as well as TUNEL staining images for Fn14, KIM-1, and TUNEL in kidney tissues from LPS-infused mice with negative control (NC) or miR-19a mimic (miR-19a) delivery. (b) Tubular damage score of kidney tissues from LPS-infused mice with negative control (NC) or miR-19a mimic (miR-19a) delivery (*n* = 8). One-way ANOVA, post hoc comparisons, and Tukey's test. V: vehicle. (c) Western blotting analyses detecting the cleavage of caspase-3 in kidney tissues from LPS-infused mice with negative control (NC) or miR-19a mimic (miR-19a) delivery. (d–f) Serum creatinine (d), blood urea nitrogen (BUN) (e), and lactate (f) levels in kidney tissues from LPS-infused mice with negative control (NC) or miR-19a mimic (miR-19a) delivery (*n* = 8). One-way ANOVA, post hoc comparisons, Tukey's test. V: vehicle.
