# Supplementary material for: MicroRNA-19a Targets Fibroblast Growth Factor-Inducible Molecule 14 and Prevents Tubular Damage in Septic AKI
Source: Anal Cell Pathol (Amst). 2020 Jul 1;2020:2894650. doi: 10.1155/2020/2894650 (PMC7349421; doi:10.1155/2020/2894650)
Supplement: Supplementary Materials — Supplemental Figure 1: histone deacetylase 3 (HDAC3) is essential for the LPS-inducible downregulation of miR-19a. (a, b) RT-qPCR detecting levels of miR-19a mRNA expression in murine MCT (a) and RAW 264.7 (b) cells stimulated with or without LPS in the presence of a pan-HDAC inhibitor trichostatin A (TSA) or a selective HDAC3 inhibitor RGFP966 (RGFP) administration. ∗∗P < 0.01 and ∗∗∗P < 0.001, one-way ANOVA, post hoc comparisons, Tukey's test. Supplemental Figure 2: repression of Fn14 and CYLD by miR-19a is independent of each other. (a) RT-qPCR determining levels of Fn14 mRNA expression in murine MCT cells transfected with empty vector (Vector) and Flag-tagged wild-type CYLD. (b) Western blotting analyses examining amount of Fn14 protein expression in murine MCT cells transfected with empty vector (Vector) and Flag-tagged wild-type CYLD. (c) Cellular ubiquitination assays detecting the levels of K48-linked polyubiquitination of Fn14 in murine MCT cells transfected with empty vector (Vector) and Flag-tagged wild-type CYLD. (d) Western blotting analyses comparing levels of CYLD protein expression in murine MCT cells with or without CYLD shRNA (sh.CYLD) transfection. (e) RT-qPCR evaluating Fn14 mRNA expression in murine MCT cells transfected with mock (Mock) and negative control (NC) as well as miR-19a mimics (miR-19a) in the presence or absence of CYLD shRNA (sh.CYLD) cotransfection. Scr: scrambled shRNA. ∗P < 0.05, one-way ANOVA, post hoc comparisons, Tukey's test. (f) Western blotting analyses assessing levels of Fn14 protein expression in murine MCT cells transfected with mock (Mock) and negative control (NC) as well as miR-19a mimics (miR-19a) in the presence or absence of CYLD shRNA (sh.CYLD) cotransfection. Supplemental Figure 3: miR-19 levels after overexpression. (a–d) RT-qPCR testing levels of miR-19a mRNA expression in murine MCT (a, c) and RAW 264.7 (b, d) cells with negative control (NC) and miR-19a mimic (miR-19a) transfection. ∗∗∗P < 0.001, two-sided [file 2894650.f1.pdf]

MicroRNA-19a targets fibroblast growth factor-inducible molecule 14 and prevents tubular damage in septic AKI

Jun Hong[1], Bang-Chuan Hu[1], Liang Xu[1], Yang Zheng[1], Zi-Qiang Shao[1], Run Zhang[1], Xiang-Hong Yang[a], Ren-Hua Sun[1], Shi-Jing Mo[1]*

[1]*Department of Intensive Care Unit, Zhejiang Provincial People's Hospital, People's Hospital of Hangzhou Medical College, Hangzhou 310014, Zhejiang, P.R.China*

Corresponding author:

*Shi-Jing Mo, MD, PhD

Department of Intensive Care Unit

Zhejiang Provincial People's Hospital

People's Hospital of Hangzhou Medical College

Hangzhou, Zhejiang 310014

P.R. China

Email: moshijing@hmc.edu.cn

Supplemental Information

Supplemental Materials and Methods

2.5. TUNEL assay

In brief, cells grown in 6-well plates were undergone the indicated treatments, incubated with the TUNEL reaction mixture for 60 min at 37˚C and stained with 5 mg/mL 4',6-diamidino-2-phenylindole (DAPI) in the dark. The reactions were terminated by rinsing three times with PBS and the number of apoptotic cells were observed and quantified using a fluorescent microscope (IX71; Olympus, Japan).

2.6. Dual-luciferase reporter (DLR) assay

Cells were seeded in triplicate in 24-well plates and allowed to settle for 24 hours. Indicated plasmids plus 1 ng pRL-TK *Renilla* plasmid were transfected into the cells using Lipofectamine 2000 Reagent (Life Technologies). Forty-eight hours after transfection, DualLuciferase Reporter Assay (Promega) was performed according to the manufacturer's instructions, as previously described[14,15]. The 3′ UTR regions of wild type and mutant Fn14 were amplified by PCR using the following primers: Fn14-WT: 5′-CGAGCTCGAAGCCTCAATCTGGGTCACAA-3′ (forward) and 5′-CCCGGGG GCATTATAGCCCCTCCGAGT-3′ (reverse). Fn14-Mut: 5′-CGAGCTCGAACTCGGAGGGGCTATAATGC-3′ (forward) and 5′-CCCGGGGGGAGATGGTTGTTTCCGTGT-3′ (reverse).

2.7. Real-time quantitative PCR (RT-qPCR)

Briefly, total RNA was isolated by Trizol (Invitrogen) and complementary DNA was synthesized with PrimeScript®RT Reagent Kit (Takana, Dalian, China) using Super Array PCR master mix (SuperArray Bioscience, USA). The sequences of the primers were as follows: Fn14 sense, 5′-GTGTTGGGATTCGGCTTGGT-3′; Fn14 antisense, 5′-GTCCATGCACTTGTCGAGGTC-3′. GAPDH sense, 5′-AGGTCGGTGTGAACGGATTTG-3′; GAPDH antisense, 5′-GGGGTCGTTGATGGCAACA-3′. Real-time PCR was then performed on an Applied Biosystems 7900HT cycler using Takana SYBR®
Primix Ex TaqTMKit (Takana, China).

2.8. Cellular ubiquitination assay

The indicated cells were lysed in radio-immunoprecipitation assay (RIPA) buffer with protease inhibitors and phosphatase inhibitor cocktail (KeyGene, China), followed by immunoprecipitated using protein A/G-sepharose beads (Cwbiotech, China) overnight at 4°C. Polyubiquitinated Fn14 was detected with an anti-K48 Ub antibody.

2.9. Western-blotting

Briefly, total protein from each sample was fractionated in 10% sodium dodecyl sulfate (SDS)-polyacrylimide gel electrophoresis and transferred to the Immobilon™ PVDF Transfer Membranes (Millipore Corporation, Billerica, MA). The membrane was then blocked in 5% bovine serum albumin (BSA) and incubated with the primary antibodies as indicated overnight, followed by incubation with HRP-linked secondary antibodies and visualization using western chemiluminscent HRP Substrate Kit (PPLYGEN, Beijing, China).

2.11. Immunohistochemical staining

In brief, the rehydrated sections or coverslips were incubated with primary antibodies at 4°C overnight, followed by incubation with horseradish peroxidase conjugated secondary antibodies for 1h. Images were imaged with a AxioVision Rel.4.6 computerized image analysis system (Carl Zeiss) and obtained using standard methods. At least three repeats were conducted for each calculation.

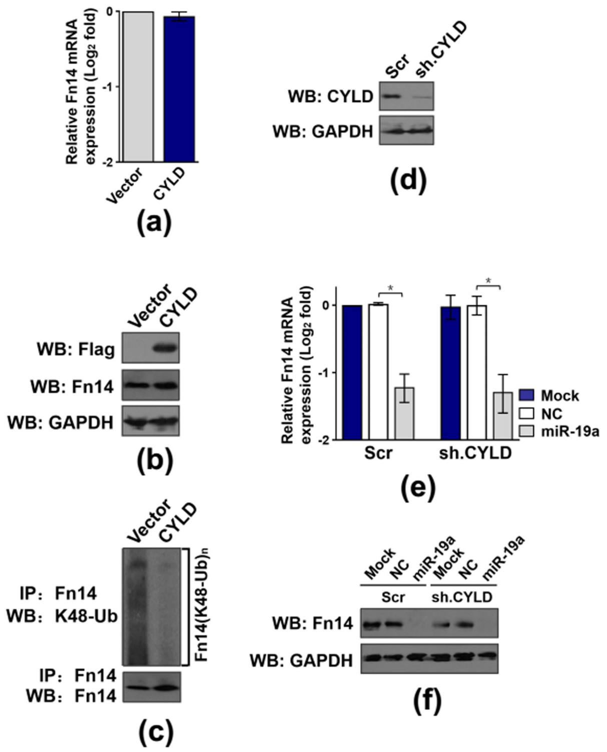

Supplemental Figure 1: Repression of Fn14 and CYLD by miR-19a is independent of each other. (a): RT-qPCR determining levels of Fn14 mRNA expression in murine MCT cells transfected with empty vector (Vector) and Flag-tagged wild-type CYLD. (b): Western-blotting analyses examining amount of Fn14 protein expression in murine MCT cells transfected with empty vector (Vector) and Flag-tagged wild-type CYLD. (c): Cellular ubiquitination assays detecting the levels of K48-linked polyubiquitination of Fn14 in murine MCT cells transfected with empty vector (Vector) and Flag-tagged wild-type CYLD. (d): Western-blotting analyses comparing levels of CYLD protein expression in murine MCT cells with or without CYLD shRNA (sh.CYLD) transfection. (e): RT-qPCR evaluating Fn14 mRNA expression in murine MCT cells transfected with mock (Mock), negative control (NC) as well as miR-19a mimics (miR-19a) in the presence or absence of CYLD shRNA (sh.CYLD) cotransfection. Scr, scrambled shRNA. *$P < 0.05$, one-way ANOVA, post hoc comparisons, Tukey's test. (f): Western-blotting analyses assessing levels of Fn14 protein expression in murine MCT cells transfected with mock (Mock), negative control (NC) as well as miR-19a mimics (miR-19a) in the presence or absence of CYLD shRNA (sh.CYLD) cotransfection.
